# Emotional Processing and Experience in Amyotrophic Lateral Sclerosis: A Systematic and Critical Review

**DOI:** 10.3390/brainsci11101356

**Published:** 2021-10-15

**Authors:** Laura Carelli, Federica Solca, Sofia Tagini, Silvia Torre, Federico Verde, Nicola Ticozzi, Monica Consonni, Roberta Ferrucci, Gabriella Pravettoni, Barbara Poletti, Vincenzo Silani

**Affiliations:** 1 Department of Neurology and Laboratory of Neuroscience, Istituto Auxologico Italiano, IRCCS, 20149 Milan, Italy; s.tagini@auxologico.it (S.T.); silviatorre.psy@gmail.com (S.T.); f.verde@auxologico.it (F.V.); n.ticozzi@auxologico.it (N.T.); b.poletti@auxologico.it (B.P.); vincenzo@silani.com (V.S.); 2 Department of Pathophysiology and Transplantation, “Dino Ferrari” Center, Università degli Studi di Milano, 20122 Milan, Italy; federica.solca@gmail.com; 3 Unità Operativa di Neurologia e Neuroriabilitazione, Istituto Auxologico Italiano, IRCCS, 28824 Piancavallo, Italy; 4 Motor Neuron Disease Centre, 3rd Neurology Unit, Clinical Neuroscience Department, Fondazione IRCCS Istituto Neurologico Carlo Besta, 20133 Milan, Italy; monica.consonni@istituto-besta.it; 5 “Aldo Ravelli” Center for Neurotechnology and Experimental Brain Therapeutics, Department of Health Sciences, International Medical School, University of Milan, 20122 Milan, Italy; Roberta.Ferrucci@unimi.it; 6 Neurology Clinic III, ASST Santi Paolo e Carlo, 20142 Milan, Italy; 7 IRCCS Ca’ Granda Foundation Maggiore Policlinico Hospital, 20162 Milan, Italy; 8 Department of Oncology and Hemato-Oncology, University of Milan, 20122 Milan, Italy; gabriella.pravettoni@unimi.it; 9 European Institute of Oncology, IRCCS, 20141 Milan, Italy

**Keywords:** amyotrophic lateral sclerosis, motor neuron diseases, emotions, alexithymia

## Abstract

Even though increasing literature describes changes in emotional processing in Amyotrophic Lateral Sclerosis (ALS), efforts to summarize relevant findings are lacking in the field. A systematic literature review was performed to provide a critical and up-to-date account of emotional abilities in ALS. References were identified by searches of PubMed, Web of Science and Scopus (1980–2021, English literature), with the following key terms: (“Amyotrophic Lateral Sclerosis” or “Primary Lateral Sclerosis” or “Motor Neuron”) and “Emotion*” and (“Processing” or “Attribution” or “Elaboration” or “Perception” or “Recognition”). Studies concerning only caregivers, pseudobulbar affect, and social cognition were excluded. Forty-one articles were included, all concerning ALS, and seven topics were identified: Emotion recognition, Emotional responsiveness, Emotional reactivity, Faces approachability rating, Valence rating, Memory for emotional materials and Alexithymia. The majority of these aspects have only been sparsely addressed. The evidence confirms altered emotional processing in ALS. The most consistent findings regard the recognition of facial expressions for negative emotions, but also alterations in the subjective responsiveness to emotional stimuli (arousal, valence and approachability), in psychophysiological and cerebral reactivity and in emotional memory, together with alexithymia traits, were reported. According to this evidence, emotional abilities should be included in the clinical assessment and therapeutic interventions.

## 1. Introduction

Recently, the literature provided consistent evidence of social cognition impairment as a cognitive manifestation of ALS, in particular in the domains of basic emotion recognition and Theory of Mind (ToM), i.e., the ability to infer others’ mental and emotional states [1].

As showed by Consonni and colleagues [2], a third dimension of cognitive impairment should be considered as a distinct pattern of non-motor manifestations in ALS patients, in addition to the recognized ALS with cognitive or behavioral impairments profiles, including language, social cognition and episodic memory. Indeed, the revised Strong criteria [3] incorporated these findings and other evidence (i.e., [4]) within the descriptions of ALS cognitive profiles.

Social cognition abilities are often considered in association with a dysexecutive syndrome and thus one of the hallmark features of frontotemporal degeneration (FTD) syndromes, according to the ALS-FTD continuum [3]. However, the relative independence of social cognition from other cognitive abilities is still a matter of debate [2].

The emotional and social cognition abilities are relevant issues in neurodegenerative disorders, according to their potential effects on patients’ quality of life and ability to engage in end-of-life decisions [5,6]. Emotional abilities help patients to maintain positive and satisfying relationships with their caregivers and relatives and sustain treatment decisions along the course of the disease [7].

Deficits in emotion processing are also associated with behavioral alterations in neurodegenerative disorders involving both cortical and subcortical diseases [8]. Moreover, in neurodegenerative disorders, the lack of emotional responsiveness and expression is also a component of apathy, related to self-awareness [9].

In the last decade, some review articles were published about social cognition and emotional processing in ALS [1,5,8,10,11,12,13,14]. The majority of studies have focused on social cognition abilities by investigating their different domains such as ToM, empathy, social perception and behavior [5].

However, only three of such reviews specifically addressed emotional processing in ALS [1,10,14]—In particular, a less recent, although more pertinent, review reports deficit in valence and arousal processing, as well as impaired facial emotion recognition of negative emotions [14]. Moreover, a possible role of disease severity and onset in emotion processing impairments and thus in the heterogeneity of findings is proposed by the Author. A successive review [10] and a meta-analysis [1] did not provide relevant improvements in this field; the review by Benbrika and colleagues [10] reported two studies supporting the presence of more alexithymic traits in ALS vs. control participants and described initial results about the longitudinal progression of emotional deficits in ALS, even if not conclusive.

The present review aims to focus on emotional processing and recognition and to summarize available findings. A critical discussion is presented, highlighting possible improvements and advances in ALS emotion research.

## 2. Materials and Methods

### 2.1. Eligibility Criteria

Randomized controlled trials, clinical trials and single case studies investigating emotional abilities in ALS and PLS were included. Participants of any age with ALS or PLS (with or without FTD) were considered. Studies including patients with only FTD without ALS/PLS or with other neurodegenerative disorders were excluded, as well as studies on healthy participants or regarding animal disease models. Primary outcome measures were behavioral responses (cognitive testing, ratings) or neuro/physiological changes (e.g., skin conductance levels and brain activity). The impact on caregivers of emotional/behavioral symptoms and pseudobulbar affect/emotional lability of patients were not considered. Moreover, studies about social cognition or ToM, if recognition/processing of emotions were not evaluated, were excluded. Report characteristics included published manuscripts (articles, book chapters) in the English language from January 1980 to March 2021. Conference proceedings were excluded, if not published as full-text articles. 

### 2.2. Information Sources

Studies were identified by searching electronic databases and scanning the article’s reference lists. The search was limited to studies published in English and applied to electronic databases of PubMed (January 1980–March 2021), Web of Science (January 1980–March 2021) and Scopus (January 1980–March 2021).

### 2.3. Search Strategy 

Studies were identified using a combination of the following terms: (“Amyotrophic Lateral Sclerosis” or “Primary Lateral Sclerosis” or “Motor Neuron”) and “Emotion*” and (“Processing” or “Attribution” or “Elaboration” or “Perception” or “Recognition”). See Supplementary Table S1 for specific text.

### 2.4. Study Selection

Titles and abstracts were screened for relevance. Full-text analysis was then performed on the selected articles. Eligibility assessment was performed independently by 2 reviewers; disagreements between reviewers were resolved by consensus.

### 2.5. Data Collection Process

We developed a data extraction sheet to summarize relevant results from the selected studies, pilot-tested it on five randomly selected included studies and refined it accordingly. One review author (LC) extracted the data from the included studies and the second author (ST) checked the extracted data. The final extraction form included: sample type and size, study designs (type of emotional abilities evaluated), main quantitative and/or qualitative outcomes related to the review topics, Level of Evidence classification (see the next paragraph). A post-hoc thematic classification of studies was created according to the type of emotional processing and instruments involved. Disagreements were resolved by discussion between the two review authors who performed the categorization; if no agreement could be reached, a third author decision (ST) was planned. Ethics committee authorization was not required as this study reviewed previously published data.

### 2.6. Risk of Bias

The quality evaluation of studies and outcomes included in the review focused on the following aspects: the Level of Evidence for the methodological quality of the studies design (based on the VII Levels rating scheme by Ackley and colleagues [15]); the representativeness of the clinical sample included (e.g., inclusion/exclusion criteria) and the selection of control participants (e.g., matching with patients according to socio-demographical variables). Moreover, more specific issues were considered, such as the type and number of emotional abilities addressed. The outcomes of this evaluation were presented in the Results section and in Supplementary Table S2 and commented on in the discussion.

## 3. Results

**Figure 1:**
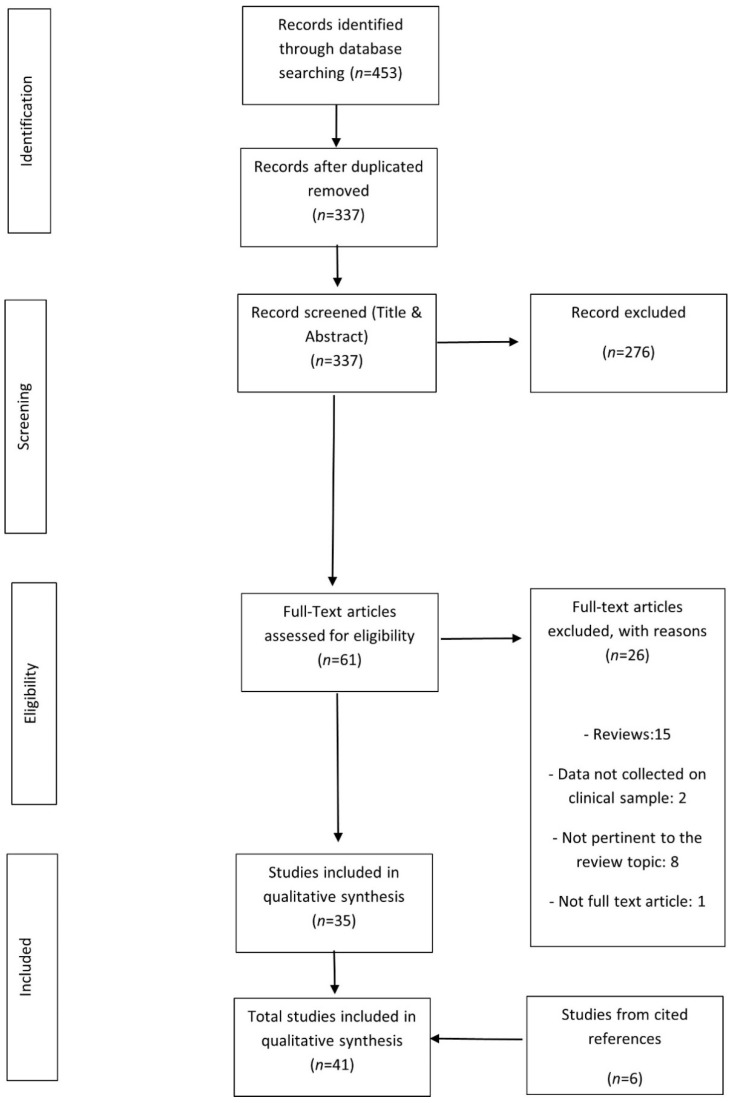
Flow chart for inclusion/exclusion of references. The flow diagram depicts the number of records identified, included and excluded, and the reasons for exclusions.

The study flow is depicted in Figure 1. A total of 41 papers met the eligibility criteria for review and their characteristics are depicted in Supplementary Table S2.

All studies include ALS patients, while no one mentions PLS or other Motor Neuron Disorders within the clinical sample. Four studies concern or include ALS/FTD patients [16,17,18,19]. One study describes a patient with anterior temporal lobe degeneration and ALS [20]. Two studies specifically select spinal ALS patients [21,22], one bulbar ALS patient [23] and one ALS type 8 patient [24].

ALS sample sizes are relatively small in most studies: only one study has more than one-hundred patients [25]; four studies have 50–100 included patients [2,16,26,27]; nineteen studies have 20–50 patients [9,18,19,24,28,29,30,31,32,33,34,35,36,37,38,39,40,41]; thirteen studies recruited 10–20 ALS patients [21,22,23,42,43,44,45,46,47,48,49,50,51]; two studies had 2–10 patients [52,53]. Two were single case studies [17,20]. 

Most studies were published in the last 10 years, in particular in 2016. Only a few studies were published before 2005. 

Reviewed studies were described according to the emotional aspects evaluated. In particular, we identified the following topics: Emotion recognition, Emotional responsiveness, Emotional reactivity, Faces approachability rating, Valence rating, Memory for emotional materials and Alexithymia (Table 1 and Supplementary Table S2).

The majority of included studies (25/41, i.e., 61%) investigated only one emotional aspect of those described in Table 1; nine studies (22%) concerned two emotional topics; five studies (12%) discussed three topics; two studies (5%) presented results about four emotional dimensions. 

The collected findings for each topic are described in detail in the following paragraphs.

### 3.1. Emotion Recognition 

The majority of reviewed studies investigated emotion recognition in ALS by means of static facial expressions of emotions [2,9,16,19,20,23,24,27,28,32,33,34,35,36,37,38,40,43,44,46,47,49,50,51,54]. Other studies evaluated emotion recognition abilities through static scenes [2,21,32,44], dynamic social vignettes [17,18,19,20,27,38], written stories [40,41], and vocal prosody [20,23,48,54].

According to facial expressions of emotions, the most adopted measurements were the Ekman 60 Faces Test-EK60-F derived from the Facial Expressions of Emotion, Stimuli and Tests-FEEST [55], the Facial Emotion Recognition Test-FERT [56], the Comprehensive Affect Testing System-CATS [57] and the Reading the Mind in the Eye Test-RME [58]. The latter was traditionally considered as a measure of affective ToM; however, according to recent findings [59], we included it among the tests of emotion recognition. 

Some studies also adopted the Ekman Caricatures Task, where the intensity of facial expressions was enhanced by altering critical facial features, or a computer-based morphing to graduate the intensity of emotional expressions, from the FEEST [55].

Heterogeneity of results was observed. Globally, only few studies failed to highlight a significantly lower performance in ALS than healthy controls-HCs [9,20,24,38,43,47,49,51]. On the contrary, most of the included studies demonstrated significant differences between patients and HCs, or abnormal scores in patients. Some study did not reported the most impaired type of emotion [2,16,25,34,40,46]. Other Authors specifically highlighted an impairment in sadness, anger, disgust or surprise recognition [19,23,27,28,31,34,36,37,44,54]; in few cases, also the recognition of happiness was involved [33]. 

With regard to the emotion recognition from static scenes, the only test involved was the Story-based Empathy Task-SET [2,30,32,44]. For such a task, all studies demonstrated a worse performance in ALS patients with respect to HCs. Globally, this impairment was at least partially independent from ALS site of onset, subtype according to Strong’s criteria, and presence/severity of executive dysfunctions. 

For dynamic social vignettes, the following materials were employed: emotional film clips [17] and the Emotion Evaluation subtest of The Awareness of Social Inference Test (TASIT-EET [60]). In the majority of studies included within this category, ALS patients showed impaired performances compared to HCs. However, two of these studies concerned ALS-FTD patients [17,18] and one additional study showed impaired performances only in the ALS-FTD subgroup [19].

Two studies carried out by Trojsi and colleagues employed written stories [40,41]. One of them failed to identify deficits at baseline but described a significant decrease for such ability at the six-months follow-up in bulbar patients [41]. Differently, the other study [40] highlighted significantly lower scores than HCs in early-stage ALS and no significant differences between bulbar and spinal patients. 

Some studies used the Aprosodia Battery [48], the CATS [20,54] and emotionally intoned neutral sentences [23] to evaluate emotion recognition from vocal prosody. Half of them reported impaired recognition of effects from emotional prosody in ALS patients compared to HCs [48,54], independently for different emotions. In 23% of bulbar patients, Zimmerman and colleagues [23] found deficits in emotional prosody recognition (scores below the 95% of CI of controls), and moderate correlations between performances at Emotional Faces and Prosody Tasks.

Only one article investigated cross-modal integration of emotional information, i.e., matching facial affect to emotional prosody [54]. At the specific CATS item inquiring emotional prosody recognition, ALS participants performed significantly more poorly than HCs. 

Thus, emotion recognition was mainly investigated by means of static facial expressions, providing quite unequivocal findings of impairment in ALS patients, primarily concerning negative emotions (sadness, anger, disgust). Emotion recognition related to other stimuli provides more sparse and heterogeneous results.

### 3.2. Emotional Reactivity

The studies inquiring about emotional reactivity adopted functional magnetic resonance imaging (MRI), electroencephalography (EEG) and psychophysiological approaches. In the former category we identified four manuscripts [22,28,50,53]. Two studies adopted emotional faces as eliciting stimuli [28,50], one unpleasant/neutral words [53] and one on pictures of social everyday life situations [22]. Globally, the studies based on functional MRI detected altered emotional processing in ALS compared to HCs, with reduced activations of cerebral structures typically involved in emotional experience (i.e., right hemisphere and limbic system) and compensatory recruitments of distinct areas in the left hemisphere, suggesting a functional reorganization in ALS. 

Kotchoubey and colleagues [52], provided evidence that in both the examined completely locked-in patients (including a participant with ALS), evoked response potentials (ERPs) to emotional vocalizations significantly differentiated between sad and joyful exclamations. A similar result was obtained with the severely paralyzed ALS patient. This result implies the ability to discriminate affective vocalizations of patients but does not provide any cues about their recognition of these effects or subjective emotional experience.

Only two studies adopted psychophysiological parameters [17,21] to measure emotional responsivity; these include: skin conductance, startle eyeblink responses, heart rate and eye movements. In the study of Liu and colleagues [17], an overall flattening of autonomic arousal with respect to stimuli eliciting both positive and negative emotions was observed. Lulé and colleagues [21] used the pictures of the International Affective Picture System (IAPS) database to elicit autonomic responses; however, no differences were observed between patients and HCs on psychophysiological parameters. The former results, to be observed, were collected on an ALS-FTD patient, while the group study did not include demented patients.

Overall, the available evidence concerning reactivity to emotional stimuli suggests altered processing and reorganizations at least according to cerebral regions and circuits involved; very limited data regarding the other biological correlates of emotional experience.

### 3.3. Emotional Responsiveness—Arousal and Elicited Emotions

In this section, we included five studies investigating the subjective experience related to emotion-eliciting stimuli, represented by static visual stimuli depicting social interactions and facial expressions [21,22], emotional film clips [42], a heterogeneous range of stimuli (i.e., reliving memories, engaging in embarrassing tasks and in conversations with parents about emotionally evocative topics) [17] and moral dilemmas [32]. Data were based on verbal ratings according to ad hoc created inventories [17,21,22,32] or more structured measurements [42]. In the longitudinal study by Kilani and colleagues [42], ALS patients experienced more joy in response to the films than HCs, whereas, at follow-up, a decline in emotional reactivity was observed in patients. The case study of an FTD-ALS patient showed flattened responsivity and responses about emotional valence evoked by emotionally eliciting stimuli, feeling no specific or nuanced emotion beyond crude positive or negative states [17]. 

Crespi and colleagues [32] demonstrated a lower rating of emotional arousal associated with moral dilemmas in both “instrumental” (i.e., the death of one person is a means to save more people) and “incidental” (i.e., the death of one person is a foreseen but unintended consequence of the action aimed at saving more people) conditions. Lulé and colleagues [21,22] reported altered arousal ratings, with a tendency to neutralize extreme pictures (calm or exciting) with respect to HCs.

Overall, these studies suggest an incipient and progressive emotional blunting in ALS.

### 3.4. Valence Attribution

This emotional dimension represents the individual’s ability to evaluate an experience considering the relative “emotional valence” (e.g., pleasantness/unpleasantness). Three articles are included in this section [21,32,45]. Verbal ratings of valence were collected regarding different emotional stimuli (words, pictures of social interactions, moral dilemmas). Two studies highlighted a tendency of ALS patients to enhance the positivity of emotional stimuli [21], by providing stronger affective ratings to positive words than to negative ones [45]. In a task requiring to rate the emotional valence of moral dilemmas (instrumental vs. incidental), no differences were observed between ALS patients and HCs [32].

### 3.5. Facial Approachability Rating

Three studies were found in this category, evaluating the rating of approachability of unfamiliar faces with neutral and emotional facial expressions [26,39,49]. While the study by Papps and colleagues failed to detect a significant difference between ALS patients and HCs [49], two successive studies by Schmolck and colleagues highlighted relevant peculiarities among ALS patients [26,39]. In the first work, a significant tendency was described in patients to rate the 10 most negative faces much more positively than controls [39]. In the more recent study [26], the authors found that 61% of ALS patients had abnormal responses, suggesting an inability to correctly recognize the threat in a given social context. Specifically, two distinct patterns were observed in patients: the “Trusters”, who evalued as more approachable those faces rated less positively by controls, and the “Suspicious Responders’ who evalued as less approachable those faces rated as very approachable by controls. The authors explain such results according to a common underlying altered amygdala functioning, leading to impairments in social judgments from faces in ALS patients.

### 3.6. Memory for Emotional Material

Four studies included within this section investigate the emotional memory effect, that is the increase in performance for emotional compared to neutral items [17,45,49,53].

Two studies showed in ALS patients the absence of the enhanced recognition memory for emotional materials (words), commonly observed in normative patterns [49,53]. Another study [45] found instead that ALS patients and HC groups did not differ significantly on measures of emotional memory, but a subgroup of patients had poor performances at an emotion recognition task and scored poorly also at other verbal memory measures. Finally, a case study examined memory of autobiographical emotional events in an ALS-FTD patient, showing preserved memories [17].

### 3.7. Alexithymia

Alexithymia refers to difficulties in emotional self-regulation. Two main forms of alexithymia were identified: the absence of the emotional experience leading to emotional cognition deficit (Type I), and the weakening in emotional cognition with sparing of emotional experience (Type II).

Only one article investigated the construct of Alexithymia in ALS patients by means of the 20-item Toronto Alexithymia Scale—TAS-20 [29]. This study revealed significantly higher mean TAS-20 total and difficulty in identifying emotions (DIF) subscores in ALS patients when compared to matched controls. The DIF subscore is related to the perception of physical sensations and awareness of their significance, i.e., to the more “emotional” ability (Type 1 Alexithymia), rather than the mentalizing components (Type 2 Alexithymia). Overall, 53.6% of patients proved to be alexithymic (TAS-20 total score) compared with just 23.3% of controls. No correlations were observed between TAS-20 scores and executive functions, while alexithymic traits correlated significantly with the gray matter volume of the prefrontal cortex, right superior temporal pole and parahippocampal gyri.

### 3.8. Relationship between Emotional and Clinical Aspects

The possible association between emotional abilities and ALS clinical aspects was heterogeneously addressed in the included studies. Below we present findings concerning emotional abilities in ALS cognitive phenotypes and their relationship with clinical features, including genotype, type of disease onset and disease progression.

Most articles did not correlate emotion deficits to patients’ cognitive phenotypes characterized according to Strong’s classification [3] or to the presence of mild to severe cognitive-behavioral alterations. Four studies specifically recruited ALS-FTD patients and compared their performance on emotional tasks with that of pure ALS patients, behavioral variant FTD (bvFTD) and/or HCs [16,17,18,19]. Unlikely ALS patients without dementia [18,19], ALS-FTD patients showed impairment at different emotion recognition tasks that were more severe than bvFTD patients [16]. A single case description confirmed the impairment in a wide range of emotional abilities in an FTD-ALS patient [17].

Some articles showed in ALS with cognitive or behavioral impairments subgroups lower performances than control participants in emotion recognition tasks, while the ALS total group was not impaired [35,36]. Differently, other studies found a similar proportion of patients with and without cognitive/behavioral deficits among those who presented impaired recognition of emotions according to facial expressions or story-based emotion recognition tests [23,31].

The possible association between executive functions and emotional abilities was investigated by ten articles [2,25,26,27,29,30,36,37,39,46,54]. About half of the studies found a significant positive relationship between those variables, in particular between executive functions and eyes/facial emotional recognition [25,37,46], cross-modal integration [54] and a social cognition composite index including RME [27]. Other studies failed to find associations between executive functions and Alexithymia [29], rating of faces approachability [39], eye/facial emotional recognition [36] and story-based emotion recognition [30]. Consistently, Consonni and colleagues found that deficits in emotion recognition and ToM did not depend on executive dysfunction in ALS [2].

No studies, but one, have examined the contribution of genetic mutations to emotional processing in ALS. The only study recruiting patients with a rare familiar form of ALS caused by a p.P56S mutation in the VAPB gene, i.e., ALS Type 8 [24], depicted a cognitive profile characterized by a prominent impairment in executive functions, with preserved abilities of facial emotion recognition.

Another clinical feature that was considered to impact emotional processing concerns the bulbar vs. spinal onset [23,30,34,40,41,42,43,46,54]. Five studies found a significantly higher involvement of emotional abilities in bulbar vs. spinal onset patients, with regard to facial and eyes emotion recognition [23,40,41,46] and story-based emotion attribution [30]. Other studies did not find any significant difference according to disease onset in eyes/facial emotion recognition or multimodal emotion processing [34,37,43,54].

Four studies adopted a longitudinal design investigating emotional responsiveness [42], facial emotion recognition [33,41], and brain responses to emotional stimuli [22]. A reduction in emotional reactivity over one year was observed [42], together with reduced insular and extrastriate visual areas activity and lower subjective arousal after six months [22]. Emotion attribution based on written stories was impaired at the six months follow-up [41]. Conversely, overt facial emotion recognition did not worsen after nine months in one study [33].

### 3.9. Risk of Bias

With reference to the study design, the majority of the included studies have a level IV of evidence (case-control studies) and three have a level VI of evidence (two single case studies and one descriptive qualitative study). Level of Evidence classification: Level I: evidence from a systematic review or meta-analysis of all relevant RCTs (randomized controlled trial) or evidence-based clinical practice guidelines based on systematic reviews of RCTs or three or more RCTs of good quality that have similar results; Level II: Evidence obtained from at least one well-designed RCT; Level III: Evidence obtained from well-designed controlled trials without randomization (i.e., quasi-experimental); Level IV: Evidence from well-designed case-control or cohort studies; Level V: Evidence from systematic reviews of descriptive and qualitative studies (meta-synthesis); Level VI: Evidence from a single descriptive or qualitative study; Level VII: Evidence from the opinion of authorities and/or reports of expert committees.

Concerning the population involved, most studies adopted the absence of FTD or major cognitive deficits as inclusion criteria for the non-demented clinical group. Only two studies also excluded patients who presented mild cognitive impairment according to a standard neuropsychological assessment [28,50]. In view of the proportion of ALS patients presenting with some degree of cognitive involvement [3], this approach to patient selection limits the representativeness of the recruited sample and the generalizability of results [3].

With regard to control participants, most studies recruited age- and education-matched healthy subjects. Seven studies did not match controls according to education [16,18,19,26,29,39,47]. Notably, among these studies, we find the one about Alexithymia, which is well known to be associated with sociodemographic variables [61]. 

According to a thematic perspective, the above-reported categorization of the reviewed studies highlights an important bias related to the paucity of studies falling in most presented categories, in particular for Alexithymia. Thus, the heterogeneity and lack of solidity of reported findings in relation to some emotion abilities severely limit the possible considerations about most topics, with the exception of emotion recognition.

## 4. Discussion

Overall, the summarized evidence confirms an altered emotion processing in ALS, as supported by different approaches and methods. In particular, the recognition of facial expressions was more deeply investigated among emotional abilities, and impairment for negative emotions (sadness, anger, disgust) was observed in several studies and across different measures. With reference to recognition of emotion expressed by vocal prosody and more dynamic and ecologic stimuli, such as video clips, the paucity of studies and heterogeneity of results lead to poor informative conclusions.

Interesting findings arise from patients’ ratings of subjective experience elicited by emotional stimuli in terms of arousal, approachability and emotions experienced. Such an approach highlights a reduced, and possibly progressive, emotional reactivity in patients, accompanied (or preceded) by a tendency to overestimate positivity and neutralize negativity of the presented stimuli. Similarly, studies about valence ratings show the tendency to enhance the positivity of emotional stimuli. 

The trend to neutralize negative stimuli might be associated with the lack of the emotional memory effect in ALS patients. In two out of three studies investigating memory for emotional materials, an absence of the enhanced recognition memory for emotional materials, commonly observed in normative populations, was highlighted. As suggested by previous research, emotionally enhanced memory relies on arousal and valence respectively involving automatic and controlled processes [62]. Taken together, the reported evidence seems to confirm a reduced reaction, and thus a less implicit reinforcement effect to emotional stimuli in ALS.

Aside from these overt changes in emotional abilities, brain activation and physiological approaches provide interesting implicit findings. In particular, abnormal lateralization of emotional processing [53] was observed in response to emotional stimuli, with an increased left hemisphere activation. Moreover, reduced or altered activation of cerebral structures typically involved in emotional experience, in particular, the limbic system, was observed in both early disease stages [50] and longitudinal observations [22].

Accordingly, the subjective experience of emotions seems altered in ALS, as suggested by the administration of the most adopted approach for alexithymia evaluation, i.e., the TAS-20 [29].

Heterogeneous and sparse findings were collected about the relationships between clinical aspects and emotional abilities. Larger efforts have been spent for the purpose of characterizing the possible relationship of emotional abilities with the disease onset (bulbar and spinal) and executive dysfunctions. However, mixed results and the absence of a clear effect of such clinical variables were reported. The few studies adopting a longitudinal design suggest the presence of a worsening in emotional processing abilities and of a cerebral spreading of the limbic involvement. 

The only reliable finding seems that ALS-FTD is related to greater severity of emotion processing impairments, while no clear considerations can be drawn for ALS with cognitive or behavioral impairments patients with respect to ALS pure disease ones. Therefore, it is not possible to establish if abnormal emotional processing in ALS is mainly related to cognitive (namely dysexecutive) or behavioral impairment.

Globally, the described studies suggest the necessity of a complex, multi-level and multi-modal, frame of interpretation of emotional processing changes in ALS, only sparsely adopted until today.

In particular, the elaboration of and reaction to emotional stimuli seem to be altered in ALS in both explicit, or self-conscious and top-down controlled, processes (as measured by self-rating and questionnaires) and implicit, or unconscious, automatic and embodied processes (i.e., cerebral and physiological activation) [63]. Moreover, in our real-life dynamic experience, people not only rely on facial but also on bodily communication. Emotions are nearly linked to actions, and the bodily expressions provide information about intentions and ongoing actions, also over a larger distance. The entire body is so involved in emotional experiences, both at the level of covert experience and of overt expression; thus, our understanding of emotional processing could be more complete if more of the bodily aspects related to the emotional experience would be included in the research. Recently, different approaches involving the body at different levels were adopted in the study of emotions in neurodegenerative disorders. The use of body expressions, aside from facial emotions, was proposed and increasingly adopted as emotion eliciting stimuli also in neurodegenerative disorders [64,65]. Moreover, different bodily information in relation to emotional stimuli, such as eye movements and facial muscle activation, can be collected and integrated with each other and with indexes of emotion recognition abilities [66,67].

The assessment of emotional abilities in ALS patients entails important implications for patients’ everyday life and for clinical practice. Affective ToM abilities, i.e., those considered in our review, seem to be associated with “mental health” quality of life [40]. Moreover, a recent review about predictors of distress in ALS showed that the only relevant factor is represented by lower levels of perceived social support [68]. Since the ability to adequately experience, recognize and respond to emotional stimuli is a constitutive aspect of social relationships, the assessment and management of emotional abilities alterations is very important for maintaining positive and supportive interpersonal exchanges. Additionally, relationships between patients and their partners along with the disease progression, in particular emotional support, intimacy and sexuality, can be affected by patients’ impairment of emotional abilities and behavioral changes [69]. The evaluation of emotion processing, social cognition and ToM in ALS may have a relevant impact in clinical settings. It would regard the possibility to assess, and support, the patients’ ability to provide a real informed consent to treatments and end-of-life decisions, which entails both cognitive, relational and affective evaluations; moreover, it could help to tailor medical communications to patients’ abilities, i.e., by enhancing non-verbal aspects or monitoring the patients’ level of comprehension [5].

As described in the “risk of bias” paragraph, the evidence included in the present review has some limitations. The quality of the studies is globally limited, with evidence provided from descriptive and sometimes narrative reports of patients. Moreover, the patient’ populations vary across studies according to demographical aspects and clinical phenotypes. From an outcome level, some important topics were not or only sparsely considered in the recruited studies, as presented in the “risk of bias” results section. Taken together, these issues do not allow to provide conclusive considerations about the different emotional aspects investigated.

## 5. Conclusions

Emotions in ALS were tested mainly on the recognition side, by means of static facial expressions of emotions. Contrarily, the investigation of patients’ own emotional experience is still less considered, as well as the relationship between self-other emotion processing and recognition. With the above-described limitations, the reported evidence confirms altered emotional processing in ALS, characterized by impaired recognition of facial expressions for negative emotions, alterations in emotional memory and in the subjective experience (arousal, valence and approachability ratings) and psychophysiological and cerebral reactivity to emotional stimuli, together with alexithymia traits. According to recent findings in other neurodegenerative disorders, emotional processing assessment in ALS should be improved and enriched. See Table 2 for suggestions about clinical and research applications of our review results.

## Figures and Tables

**Table 1:** Description of emotional variables, tests and references.

Emotion Variable	Emotional Test or Stimuli	References
**Emotion recognition (ER):** Identification of emotional states with attribution of labels (e.g., joy, sadness, disgust)		
Static facial expressions (SF)	FEEST (Ekman 60 Faces test, Ekman Caricatures task and Morphing); FERT; CATS; RME	[2,9,16,19,20,23,24,27,28,32,33,34,35,36,37,38,40,43,44,46,47,49,50,51,54]
Static scenes (SS)	SET	[2,21,33,45]
Written stories (WS)	EAT	[40,41]
Dynamic social vignettes (DS)	TASIT (emotion evaluation subtest); emotional film clips	[17,18,19,20,27,38]
Emotional prosody (PR)	CATS; aprosodia battery	[20,23,48,54]
Cross-modal integration (CI)	CATS	[54]
**Emotional reactivity:** Physiological, muscular and brain activity correlates of emotional stimulations		
Coded facial expressions (CF)	variety of subjective/objective emotional stimuli	[17]
Physiological data-PA (SC, EMG, GSR, HR, EM)	IAPS; variety of subjective/objective emotional stimuli	[17,21]
Brain activation data-BR (fMRI, EEG)	POFA; FEEST; unpleasant/neutral words; IAPS; emotional vocalizations	[22,28,50,52,53]
**Emotional responsiveness (ER):** Subjective reporting of emotional activation	IAPS; emotional film clips, variety of subjective/objective emotional stimuli; moral dilemmas.	[17,21,22,32,42]
**Approachability attribution (AA):** Subjective rating of friendliness of presented emotional faces.	60 images of faces (from Adolphs and colleagues, 1998)	[26,39,49]
**Valence attribution (VA):** Subjective rating of the affective quality related to the attractiveness/averseness of presented stimuli	IAPS; moral dilemmas; Brierley–Medford sentences; Phelps words	[21,32,45]
**Alexithymia (AX)**: Difficulty in identifying and describing emotions and feelings	TAS-20	[29]
**Memory for emotional material (ME):** Encoding and retention of information related to emotional stimuli/experiences	Brierley–Medford sentences; Phelps words; emotional/neutral sentences; unpleasant/neutral words; autobiographical material	[17,45,49,53]

Legend: EK60F = Ekman 60-Faces test; RME = Reading the Mind in the Eyes test; TASIT = The Awareness of Social Inference Test; EAT = Emotion Attribution Task; FEEST = Facial Expressions of Emotions Stimuli and Test; SET = Story-based Empathy Task; CATS = Comprehensive Affect Testing System; SEA = Social Cognition and Emotional Assessment; FERT = Facial Emotion Recognition test; TAS-20 = Toronto Alexithymia Scale; IAPS= International Affective Picture System; POFA = Pictures of Facial Affect; SC = skin conductance; GSR = galvanic skin response; HR = heart rate; EM = eye movements; EMG = electromyography.

**Table 2:** Bullet points about clinical and research issues.

Research Issues
To adopt an integrated multi-level assessment approach to emotional abilities
To adopt an embodied perspective addressing different aspects of the bodily experience in relation to emotional stimuli (e.g., eye movements, muscular activity, psycho-physiological activation)
To address both self- and other emotion processing abilities
To clarify the unsolved topics concerning clinical (genotype and phenotype) issues in relation to emotional processing
**Clinical Issues**
To evaluate emotional abilities during routinary neuropsychological and clinical visits
To consider the patients’ emotional abilities for the purpose of tailoring and adapting the management of therapeutic interventions and end-of-life decisions
To define and implement therapeutic interventions to help patients and their relatives in managing issues arising from emotional processing impairments

## Data Availability

Data are contained within the article or supplementary material. The data presented in this study are available in Supplementary Tables S1 and S2.

## Supplementary Materials

The following are available online at https://www.mdpi.com/article/10.3390/brainsci11101356/s1, Table S1: Literature search strategy text; Table S2: Summary of included studies and main findings.
