# Supplementary material for: Emotional Processing and Experience in Amyotrophic Lateral Sclerosis: A Systematic and Critical Review
Source: Brain Sci. 2021 Oct 15;11(10):1356. doi: 10.3390/brainsci11101356 (PMC8534224; doi:10.3390/brainsci11101356)
Supplement: Supplementary file 1 [file brainsci-11-01356-s001.zip › supplementary files/supplementary table 1.pdf]

PUBMED
((Amyotrophic lateral sclerosis[Title/Abstract]) OR (Primary lateral sclerosis[Title/Abstract]) OR (Motor neuron[Title/Abstract])) AND ((Emotion*[Title/Abstract]) AND ((Processing[Title/Abstract]) OR (Recognition[Title/Abstract]) OR (perception[Title/Abstract]) OR (Elaboration[Title/Abstract]) OR (attribution[Title/Abstract])))
SCOPUS
TITLE-ABS-KEY (("Amyotrophic lateral sclerosis" OR "Primary lateral sclerosis" OR "Motor neuron") AND (emotion* AND (processing OR recognition OR perception OR elaboration OR attribution)))
Web Of Science
AB=(((Amyotrophic AND lateral AND sclerosis) OR (primary AND lateral AND sclerosis) OR (motor AND neuron)) AND (Emotion* AND (processing OR recognition OR perception OR attribution OR elaboration)))
