# Supplementary material for: Emotional Processing and Experience in Amyotrophic Lateral Sclerosis: A Systematic and Critical Review
Source: Brain Sci. 2021 Oct 15;11(10):1356. doi: 10.3390/brainsci11101356 (PMC8534224; doi:10.3390/brainsci11101356)
Supplement: Supplementary file 1 [file brainsci-11-01356-s001.zip › supplementary files/supplementary table 2.pdf]

Article	Sample Type	Emotion Variables	Main Results	LOE
Ahmed et al. 2020	59 ALS-FTD and 39 bvFTD pts	SF	Both motor and cognitive onset ALS-FTD showed reduced emotion processing and exhibited greater motor cortex and dorsal lateral prefrontal cortex atrophy than bvFTD.	VI
Crespi et al. 2020	28 ALS pts and 36 HCs	SF, SS, VA, ER	EK60F or SET scores were impaired or borderline in half of the patient sample. HCs showed higher emotional arousal ratings for both incidental and instrumental conditions compared to ALS patients, which showed similar levels of emotional engagement in both conditions.	IV
Lillo et al. 2020	21 ALS pts, 20 bvFTD pts and 21 HCs	SF	ALSci underperformed HCs in the FERT and Mini-SEA. No significant difference between the cognitively unimpaired ALS and HCs, and between ALSci and bvFTD groups in FERT and Mini-SEA.	IV
Vonk et al. 2020	67 years old pts with anterior temporal lobe degeneration	PR, DS, SF	TASIT Social Inference and Emotion Evaluation tasks were significantly impaired at the first visit and slightly declined over the course of the years. Basic emotion processing (CATS) remained relatively preserved.	IV
de Alcantara et al. 2019	22 ALS type 8 pts and 33 HCs	SF	No statistical difference between groups in facial emotions recognition.	IV
Martins et al. 2019	21 ALS pts and 25 HCs	SF	ALS performed worse than HCs in the recognition of sadness.	IV
Yunusova et al. 2019	17 ALS pts and 12 HCs	SF	No differences at the emotion recognition and affective TOM tests between ALS pts and HCs.	IV
Benbrika et al. 2018	28 ALS pts and 30 HCs	AX	ALS pts had higher total score and DIF sub-score of the TAS-20 than HCs controls.	IV
Andrews et al. 2017	33 ALS pts and 22 HCs	SF, PR, CI	Lower performances were observed in ALS at the complex identification of facial emotions, identifying emotion from voice prosody, and matching facial affect to emotional prosody.	IV
Gillingham et al. 2017	20 ALS pts and 36 HCs	SF	At the baseline, ALS participants made more errors perceiving emotional expressions only for the happy faces. At the follow-up, ALS pts improved depicting an angry emotion compared to HCs.	IV
Radakovic et al. 2017	30 ALS pts/caregivers dyads and 29 HCs/informants dyads	SF	No differences were detected between ALS pts and HCs in emotion recognition and emotional intensity rating on the EK60F. Self-rated emotional apathy was significantly correlated with emotional recognition at the EK60F.	IV
Trojsi et al. 2017	21 ALS pts and 15 HCs	WS	At baseline, no differences between ALS pts and HCs; at the six-months follow-up, ALS-B exhibited a significant impairment of both affective and cognitive ToM subcomponents, whereas the ALS-L group showed significant impairment of the cognitive subcomponent alone.	IV
Aho-Ozhan et al. 2016	30 ALS pts and 29 HCs. A subgroup of 15 ALS and 14 HCs made fMRI	SF, BR	ALS patients showed decreased brain and behavioral responses in processing of disgust and fear and an altered brain response pattern for sadness.	IV
Burke et al 2016	106 ALS pts and 50 HCs	SF	HCs > ALS pts with executive impairment and single executive deficits. HCs = ALS pts with no cognitive abnormalities. ALS pts without cognitive deficits = ALS pts but > ALS patients with multi-domain executive impairment. ALS pts with single executive deficits = ALS pts with multi-domain executive impairment.	IV
Consonni et al. 2016	71 ALS pts	SF, SS	SET emotion attribution: 12,7% subnormal performances; Ekman: 25,8% subnormal performances. An additional third cognitive cluster loaded on social cognition, language and memory tests and accounted for	IV

			24% of the patients.	
Crespi et al. 2016	22 ALS pts and 55 HCs (19 ALS and 20 HCs with DTI)	SS	SET emotion attribution condition was the only one impaired in ALS pts, with 6 out of 13 patients (2 ALSbi, 1 ALSci, 3 pure ALS) performing equal to or below the 5th percentile of the HCs' scores.	IV
Oh et al. 2016	24 ALS pts and 24 HCs	SF	Significant differences were observed between ALS pts and HCs in the percentages of correct answers for anger, disgust, and surprise.	IV
Trojsi et al. 2016	22 ALS pts and 15 HCs	SF, WS	ALS pts performed significant worse than HCs on EAT and RME. ALS-B had lower scores than ALS-S on RME, with no significant differences on EAT.	IV
Jelsone-Swain et al. 2015	19 ALS pts and 20 HCs	SF	No differences between ALS and HC at RME. However, patients who performed better at RME had a pattern of cortical activation associated with better action understanding performance.	IV
Watermeyer et al. 2015	55 ALS pts and 49 HCs	SF, DS	No differences were detected between ALS pts and HCs at RME and TASIT. ALS-related executive dysfunction was the main predictor of social cognition performance.	IV
Woolley et al. 2015	305 pts with neurodegeneration (22 bvFTD/ALS and 6 ALS) and 90 HCs	DS	bvFTD/ALS pts were impaired at recognizing disgust compared with HCs. Subjects with disgusting behaviors were significantly more impaired at recognizing disgust and other emotions.	IV
Cerami et al. 2014	20 ALS pts and 56 HCs.	SS	Significantly reduced performance were revealed in ALS for emotion attribution; these abilities were significantly positively correlated with GM density in the right fronto-insular and anterior cingulate cortex.	IV
Crespi et al. 2014	13 ALS pts and 14 HCs	SF	A significant decline was observed in negative emotions identification (anger and disgust) in patients compared to HCs.	IV
Savage et al. 2014	29 ALS (16 FTD ALS and 13 ALS) pts, 25 bvFTD pts and 30HCs	SF, DS	Impaired recognition of anger, disgust, fear, and sadness in bvFTD and FTD-ALS.	IV
Passamonti et al. 2013	11 ALS pts and 12 HCs	BR	In ALS patients, greater activation was detected in a series of PFC areas and altered left amygdala-PFC connectivity.	IV
Staios et al. 2013	35 ALS pts and 30 HCs	SF, DS	At the TASIT, no significant difference was observed between ALS pts and HCs in the ability to recognize emotions (positive or negative).	IV
Cuddy et al. 2012	19 ALS pts and 19 HCs	VA, ME	ALS pts gave higher affective ratings to positive, compared to negative words. Although ALS pts and HCs did not differ significantly on measures of emotional memory, a subgroup of patients performed poorly on an emotional recognition task.	IV
Lillo et al. 2012	20 ALS pts, 20 bvFTD and 20 HCs	SF	Modified Ekman Test: Controls >ALS >bvFTD. Rasch Analysis: Ekman test is among measures that best captured the ALS-FTD continuum.	IV
Schmolck et al. 2012	91 ALS pts and 78 HCs	AA	While 65.4% of HCs participants were "Conventional Responders", 62.6% of ALS pts were either "Trusters" or "Suspicious Responders".	IV
Cavallo et al. 2011	15 ALS pts and 21 HCs	SF	RME: no significant differences between ALS and HCs or between bulbar and not bulbar ALS pts.	IV

Girardi et al. 2011	14 ALS pts and 20 HCs	SF	FEEST: ALS pts recognized significantly fewer emotions than HCs. RME: the number of correct responses tended towards a significant difference in ALS pts compared with HCs	IV
Meier et al. 2010	18 ALS pts and 18 HCs	PR	ALS group was significantly less able to identify emotional expression by prosody than HCs, but had no difficulty discriminating between different emotional expressions.	IV
Palmieri et al. 2010	9 ALS pts and 10 HCs	BR, ME	ALS pts showed an abnormal lateralization in emotional processing, mainly for unpleasant words (increased left hemisphere and reduced right hemisphere activation). ALS pts did not show enhanced memory for unpleasant versus neutral words.	IV
Liu et al. 2009	53 year old man with FTLD and later presentation of ALS	PA, DS, ME, ER	Diminished emotional reactivity. Difficult in identifying sexual arousal and disgust. Less impaired on emotional memory (autobiographical).	IV
Kotchoubey et al. 2009	27 pts with severe disorders of consciousness, 3 pts with severe motor disability (globally 2 ALS) and 16 HCs	BR	In both completely locked-in patients (including ALS), ERPs significantly differentiated between sad and joyful exclamations. A similar result was obtained with the severely paralyzed ALS patient.	IV
Lulé et al. 2007	13 spinal onset ALS pts, 15 HCs and 6 tetraplegic patients	VA, BR, ER	Increased brain response in the right supramarginal area and reduced brain response in extrastriate visual areas in ALS. Follow-up: reduction of brain responses in the anterior insula, correlated with subjective arousal.	IV
Schmolk et al. 2007	26 ALS pts, 26 HCs and 14 pts with congestive heart failure	AA	ALS pts rated the 10 most positive faces the same as HCs, but rated the 10 most negative faces much more positively.	IV
Zimmerman et al. 2007	13 bulbar ALS pts and 12 HCs	SF, PR	Significantly lower performances in ALS pts than HCs in emotion recognition (sadness, disgust and surprise). No significant differences in the prosody recognition task, with only lower performance in surprise.	IV
Lulé et al. 2005	12 ALS pts and 18 HCs	VA, PA, ER	Calm and neutral stimuli were rated as more arousing by patients, but the more arousing a picture was, the lower was the level of individual arousal of patients compared with HCs. No major differences between patients and controls concerning physiological responses to emotional stimuli.	IV
Papps et al. 2005	19 ALS pts and 20 HCs	SF, AA, ME	No significant differences in facial emotion recognition and in 'trustworthiness' or 'approachability' ratings; absent in ALS pts the normative pattern of enhanced recognition memory for emotional words.	VI
Kilani et al. 2004	19 ALS pts and 19 HCs	ER	ALS pts experienced more joy in response to the films than HCs. Decline in emotional reactivity between M0 and M12	IV

Legend: LOE=Level of Evidence; Pts=patients; HCs=healthy controls; ALS=Amyotrophic Lateral Sclerosis; ALS-B=bulbar-onset ALS; ALS-S=spinal-onset ALS; ALSci=ALS with cognitive impairment; ALSbi=ALS with behavioral impairment; FTD=frontotemporal dementia; bvFTD=frontotemporal dementia-behavioral variant; SF=static facial expressions; SS=static scenes; WS=written stories; DS=dynamic social vignettes; VA=Valence attribution; AA=approachability attribution; PR=emotional prosody; PA=physiological activation; BR=brain activation; ER=emotional responsiveness; AX=Alexithymia; ME= memory; TOM=Theory of Mind; EK60F=Ekman 60-Faces test; RME=Reading the Mind in the Eyes test; TASIT=The Awareness of Social Inference test; EAT=emotion attribution task; FEEST=Facial expressions of emotions stimuli and test; SET=Story-based Empathy task; CATS=Comprehensive Affect Testing System; SEA=Social Cognition and Emotional Assessment; FERT=Facial Emotion Recognition test; TAS-20=Toronto Alexithymia Scale; DIF=difficulty identifying feelings TAS-20 factor; ERP=evoked response potential; PFC=prefrontal cortex; GM=grey matter.
